# The Therapeutic Implications of Tea Polyphenols against Dopamine (DA) Neuron Degeneration in Parkinson’s Disease (PD)

**DOI:** 10.3390/cells8080911

**Published:** 2019-08-16

**Authors:** Zhi Dong Zhou, Shao Ping Xie, Wuan Ting Saw, Patrick Ghim Hoe Ho, Hong Yan Wang, Lei Zhou, Yi Zhao, Eng King Tan

**Affiliations:** 1 Department of Research, National Neuroscience Institute, Singapore 308433, Singapore; 2 Signature Research Program in Neuroscience and Behavioral Disorders, Duke-NUS Medical School, Singapore 169857, Singapore; 3 Ocular Proteomics Laboratory, Singapore Eye Research Institute, Singapore 169856, Singapore; 4 Singapore Department of Ophthalmology, Yong Loo Lin School of Medicine, National University of Singapore, Singapore 119077, Singapore; 5 Ophthalmology and Visual Sciences Academic Clinical Research Program, Duke-NUS Medical School, Singapore 169857, Singapore; 6 Department of Neurology, Singapore General Hospital, Singapore 169608, Singapore

**Keywords:** dopamine, neurodegeneration, polyphenols, Parkinson’s disease, anti-PD therapy

## Abstract

Accumulative evidence indicated that the pathologically accumulated metal ions (iron species and Mn^3+^) and abnormally up-regulated monoamine oxidase B (MAOB) activity induced oxidation of endogenous dopamine (DA) can lead to mitochondria impairment, lysosome dysfunction, proteasome inhibition, and selective DA neuron vulnerability, which is implicated in the pathogenesis of Parkinson’s disease (PD). The DA oxidation can generate deleterious reactive oxygen species (ROS) and highly reactive DA quinones (DAQ) to induce DA-related toxicity, which can be alleviated by DA oxidation suppressors, ROS scavengers, DAQ quenchers, and MAOB inhibitors. On the other hand, the nuclear factor erythroid 2-related factor 2 (Nrf2)-Keap1 and Peroxisome proliferator-activated receptor gamma coactivator 1-alpha (PGC-1α) anti-oxidative and proliferative signaling pathways play roles in anti-oxidative cell defense and mitochondria biogenesis, which is implicated in DA neuron protections. Therefore, agents with capabilities to suppress DA-related toxicity including inhibition of DA oxidation, scavenge of ROS, detoxification of DAQ, inhibition of MAOB, and modulations of anti-oxidative signaling pathways can be protective to DA neurons. Accumulative evidence shows that tea or coffee consumptions and smoking are related to deceased PD prevalence with unknown mechanisms. In this study, we investigate the protective capabilities of tea polyphenols and other PD relevant agents to inhibit DA-related toxicity and protect against environmental or genetic factors induced DA neuron degeneration in vitro and in vivo. We find that tea polyphenols can significantly suppress DA-related toxicity to protect DA neurons. The tea polyphenols can protect DA neurons via inhibition of DA oxidation, conjugation with DAQ, scavenge of ROS, inhibition of MAOB, and modulations of Nrf2-Keap1 and PGC-1α anti-oxidative signaling pathways. The tea polyphenols with more phenolic hydroxyl groups and ring structures have stronger protective functions. The protective capabilities of tea polyphenols is further strengthened by evidence that phenolic hydroxyl groups can directly conjugate with DAQ. However, GSH and other sulfhydyl groups containing agents have weaker capabilities to abrogate DA oxidation, detoxify ROS and DAQ and inhibit MAOB; whereas nicotine (NICO) and caffeine (CAF) can only modulate Nrf2-Keap1 and PGC-1α pathways to protect DA neurons weakly. The tea polyphenols are identified to protect against overexpression of mutant A30P α-synuclein (α-syn) induced DA neuron degeneration and PD-like symptoms in transgenic Drosophila. Based on achievements from current studies, the excellent and versatile protective capabilities of tea polyphenols are highlighted, which will contribute and benefit to future anti-PD therapy.

## 1. Introduction

Parkinson’s disease (PD) is one of the most common incurable neurodegenerative diseases all over the world. Globally, PD affects about 1% of adults older than 60 years. The debilitating nature and morbidity of the disease presents significant healthcare, social, emotional, and economic issues [1]. PD is characterized with progressive dopaminergic neuron degeneration and Lewy body formation in affected PD brain [2]. The progressive degeneration of dopaminergic neuron in PD brain leads to dramatic decrease of dopamine (DA) level in the Substantia Nigra (SN), triggering onset of PD clinical symptoms. So far no drugs have been developed to alleviate the progressive DA neuron degeneration in PD and the pathogenesis of PD is still under debate. Accumulative evidence suggests that oxidation of endogenous DA can be a pathological factor, leading to mitochondria impairment, proteasome inhibition, lysosome dysfunction, and DA neuron vulnerability [3,4,5,6]. Furthermore, it is demonstrated that, compared with small molecular reactive oxygen species (ROS), DA quinones (DAQ) derived from DA oxidation play more important pathological roles in DA-related toxicity to DA neurons, as DAQ can irreversibly conjugate with functional proteins, leading to protein misfolding, inactivation, and aggregation [4,6]. However, agents with sulfhydryl groups, such as glutathione (GSH) or N-acetyle-cysteine (NAC) to competitively conjugate with and detoxify reactive DAQ, are implicated in protections of DA neuron in anti-PD therapy [7,8]. The DA oxidation can also be catalyzed by monoamine oxidase-B (MAOB) localized in the mitochondria membrane [9]. It is identified that the expression and activity of MAOB, but not monoamine oxidase-A (MAOA), is significantly enhanced in PD brains [10]. The pathologically elevated activity of MAOB in PD brains will catalyze DA oxidation to generate deleterious H_2_O_2_, which aggravates oxidative stress, contributing to progressive DA neuron degeneration in PD [11]. The inhibition of MAOB can up-regulate DA level, which can alleviate motor symptoms of PD patients [12]. Furthermore, the inhibition of MAOB can be neuroprotective to DA neurons in PD brains [13]. Therefore, agents which inhibit DA oxidation, scavenge ROS, detoxify DAQ, and inhibit MAOB can be significant to anti-PD therapy. On the other hand, the protective nuclear factor erythroid 2-related factor 2 (Nrf2)-Keap1 and Peroxisome proliferator-activated receptor gamma coactivator 1-alpha (PGC-1α) anti-oxidative and proliferative signaling pathways play roles in modulation of anti-oxidative cell defense and mitochondria biogenesis [14,15]. Modulation of Nrf2-Keap1 pathway can enhance the expressions of a group of cytoprotective genes, including anti-oxidative and anti-inflammatory genes as well as transcription factors involved in mitochondrial biogenesis [16]. Activations of Nrf2 by chemical modulators or genetic manipulations are demonstrated to alleviate PD-related agents, such as MPP+, Rotenone, and H_2_O_2_, as well as genetic factors induced DA neuron loss in vitro and in vivo [17]. The PGC1α is a regulator of mitochondrial biogenesis genes that simultaneously promotes expression of many genes to protect against oxidative stress [18]. The PGC-1α is proposed to be a potential therapeutic target for PD therapy [19]. PGC1α knockout mice show aggravated susceptibility to DA neuron loss under MPP+ challenge, whereas overexpression of PGC1α is demonstrated to protect DA neurons against oxidative stress in vitro [18]. Therefore, factors to modulate Nrf2-Keap1 and PGC1α signaling pathways can protect DA neurons against DA-related toxicity.

Accumulative evidence indicates that smoking and drinking of coffee or tea are associated with a lower risk of PD [20,21,22]. Among numerous chemicals in smoke, the neuroprotective roles of nicotine (NICO) are significant, as it is identified to protect DA neurons and alleviate symptomatic in PD patients [23]. The caffeine (CAF), one of the major components of coffee, are verified to be neuroprotective in clinical studies and various PD models [24,25]. Teas are rich sources of multiple polyphenols, the most abundant being epigallocatechin-3-gallate (EGCG) and theaflavins (TF) in green tea and black tea respectively. The neuroprotective effects of tea polyphenols have been validated in various PD models [26,27]. So far multiple underlying mechanisms have been proposed for reduced PD risk by smoking and consumption of coffee or tea. NICO is reported to protect DA neurons via stimulation of nicotinic acetylcholine receptors (nAChRs), inhibition of monoamine oxidase (MAO), induction of neurotrophic factors and suppression of α-synuclein (α-syn) aggregation [28,29,30,31]. CAF is an A2A receptor antagonist and a potential monoamine oxidase B (MAOB) inhibitor [32]. CAF can inhibit nitric oxide (NO) generation, suppress α-syn protein aggregation and enhance the proliferative PI3K/Akt signaling pathway [33,34,35]. Tea polyphenols are supposed to protect DA neurons via multiple mechanisms, including anti-oxidation, iron chelating, anti-inflammation, anti-aggregation, modulations of functions and biogenesis of mitochondria, regulation of autophagy and cell death pathways, activation of cell surface receptor, and modulation of multiple proliferative signaling pathways [26,36,37]. However, so far the detailed neuron protective mechanisms of NICO, CAF, and tea polyphenols are still under debates and need further validations.

In this study, the multiple protective capabilities of various tea polyphenols, CAF, NICO, and other PD-relevant agents against DA-related toxicity are investigated. Six tea polyphenols with different numbers of phenol ring structures and hydroxyl groups are studied, including Garlic acid (GA, one phenol ring and three hydroxyl groups), epigallocatechin (EGC, three phenol rings and six hydroxyl groups), epicatechin-3-gallate (EGG, four phenol rings and seven hydroxyl groups), EGCG (four phenol rings and eight hydroxyl groups), TF (six phenol rings and nine hydroxyl groups) and Tannic acid (TA, ten phenol rings and twenty five hydroxyl groups) (detailed structure features are summarized in Table 1 and illustrated in Supplementary Figure S1). The protections of DA neurons in PD models against DA-related toxicity including anti-oxidative capacity, inhibition of DA oxidation, detoxification of deleterious by-products of DA oxidation, inhibition of MAOB, potential modulations of protective Nrf2-Keap1 and PGC-1α signaling pathways by tea polyphenols and other agents are investigated. Our results show that, among agents studied, tea polyphenols have the most excellent and versatile capabilities to protect against DA neuron degeneration in vitro and in vivo. We identify that tea polyphenols can protect against DA-related toxicity via inhibition of DA oxidation, conjugation, and detoxification of DAQ, scavenge of ROS, inhibition of MAOB, and modulations of Nrf2-Keap1 and PGC-1α anti-oxidative signaling pathways. The protective capabilities of tea polyphenols are dependent on the amounts of ring structures and phenolic hydroxyl groups of tea polyphenols. Furthermore, in the current study, we identify for the first time that phenolic hydroxyl groups can directly react and conjugate with DAQ. However, GSH, NAC, and L-cys have weaker protections to abrogate DA oxidation, detoxify ROS and DAQ and inhibit MAOB; whereas NICO and CAF can modulate Nrf2-Keap1 and PGC-1α pathways to protect DA neurons weakly. The underlying protective mechanisms of tea polyphenols and other agents and potential implications in PD pathogenesis and therapy are highlighted and discussed.

## 2. Materials and Methods

### 2.1. Materials

Acetonitril, DA, EDTA, GSH, NAC, l-cysteine (l-cys), HEPES, FeCl_3_, Manganese (III), pyrophosphoric acid, sodium pyrophosphate, Tris-HCl, tyrosinase (Tyro, from mushroom), mannitol (MAN), α-Tocopherol (VE), ascorbic acid (VC), NICO, CAF, and tea polyphenols were all purchased from Sigma-Aldrich Pte., Ltd. (St. Louis, MO, USA), except specifically described. Other chemicals and equipments used were all purchased from companies as indicated.

### 2.2. Plasmids and Constructs

The wild type (WT) and mutant 5 × antioxidant response element (ARE) elements were synthesized by Integrated DNA Technologies, PTE Ltd. (Singapore). The synthesized WT and mutant 5×ARE elements are PCR amplified and cloned into pGL3-promoter vector (WT and mutant ARE-pGL3-promoter vectors) between KpnI and NheI restriction enzyme sites using infusion cloning kit (Takara Bio Inc., Kusatsu, Shiga Prefecture, Japan) respectively. Detailed sequence of constructs map is illustrated in Supplementary Figure S3. The rat tyrosine hydroxylase (TH) expression vector is a gift from Dr Zhang Xiao Dong (Duke-NUS Medical School, Singapore). The TH shRNA plasmids for human (TRCN vectors) were purchased from Open Biosystem. The WT PGC-1α promoter 2kb luciferase vector (Plasmid #8887) and PGC-1α promoter luciferase ΔCRE vector (Plasmid #8888) are purchased from Addgene (Watertown, MA, USA) deposited by Dr Bruce Spiegelman [38]. The WT PGC-1α promoter 2kb luciferase vector is cloned with 5′ flanking sequence of mouse PGC-1α gene PCR amplified between +78 and −2533 with respect to the transcriptional start site, while the PGC-1α promoter luciferase delta CRE vector is cloned with the same fragment but with mutation of the CRE site between −146 and −129 from TGACGTCA to TAGATCTA. The pcDNA3-EGFP vector (Addgene plasmid 13031) developed by Dr. Doug Golenbock and purchased from Addgene was used as a control vector.

### 2.3. Cell Lines and Cell Viability Studies

Human HEK293T and SH-SY5Y cells were maintained in high glucose DMEM supplemented with 10% (*v*/*v*) fetal bovine serum (FBS) (Gibco, Waltham, MA, USA) and 1% (*v*/*v*) penicillin-streptomycin (Gibco). Dopaminergic neuronal PC12 cells were grown in high glucose DMEM supplemented with 5% (*v*/*v*) fetal bovine serum (FBS) and 10% (*v*/*v*) horse serum (HS) (Gibco). All culture media also contained 1 mM sodium pyruvate and 1% (*v*/*v*) penicillin-streptomycin. After cells were exposed to different concentration of toxins (Mn^3+^, H_2_O_2_ and AM) in the presence or absence of different concentration of protective agents for different time duration, cell viability were analyzed with calcein-AM-Hoechst dual fluorescence cell viability assay.

### 2.4. Aminochrome (AM) Preparation

AM, a cyclized DAQ, was prepared through catalysis of DA by Tyro [6]. Usually, 1 mM AM was produced by reaction of 1 mM DA (dissolved in distilled water) with 100 unit Tyro (1 unit/μL, 2000 unit/mg, MW ~125,000, freshly prepared in distilled water) in 1 mL distilled water for about 5 min at room temperature with constant shaking. Then different concentration of AM could be diluted and be used for experiments.

### 2.5. Inhibition of DA Oxidation by Various Protective Agents

The oxidation of 100 μM DA in distilled water is induced by 400 μM Mn^3+^, 10 unit Tyro, or 200 μM Fe^3+^ respectively in the presence or absence of different concentration of CAF, NICO, VC, VE, GSH, NAC, L-cys, GA, EGC, ECG, EGCG, TF, and TA for 3 min at room temperature before HPLC analysis of DA content in solutions.

### 2.6. ABTS Cation Decolorization Assay

The monitoring of 2,2′-azino-bis(3-ethylbenzothiazoline-6-sulfonic acid) diammonium salt (ABTS) radical scavenging activity of chemicals was measured by the ABTS cation decolorization assay as described by Re et al. with some modifications [39]. The ABTS radical cation (ABTS^•+^) was produced by reaction of 7 mM stock solution of ABTS with 2.45 mM potassium persulfate and allowing the mixture to stand in dark at room temperature for 12 h before use. The ABTS^•+^ solution was diluted with methanol to give an absorbance of 0.7 ± 0.01 at 734 nm. Chemicals in solutions (1 mL) were allowed to react with 2 mL of the ABTS^•+^ solution and the absorbance was measured at 734 nm after 1 min. The group in the absence of any reductants was set as control group. The results were expressed as % of control at OD 734 nm.

### 2.7. Conjugation of DAQ with Peptides

3 peptides containing 30 amino acids are synthesized by Pepmic Co.; Ltd. (Suzhou, China). The peptide G “HGKKQDNRGQEGGEDGDDREGGGKGNEGQD” does not containing any sulfhydryl or hydroxyl residue groups. The peptide S “HGKKQDNRSQESGEDGDDREGSGKSNESQD” replaces 5 glycine residues with serine residues, containing 5 non-phenolic hydroxyl groups. The peptide Y “HGKKQDNRYQEYGEDGDDREGYGKYNEYQD” replaces 5 glycine residues with tyrosine residues, containing 5 phenolic hydroxyl groups. Peptides G, S, and Y are synthesized, verified by HPLC and LC-MS-MS analysis (Supplementary Figure S4). To conjugate DAQ directly with peptides, 1.5 mM peptides are incubated in 1× PBS buffer in the presence or absence of 6 mM DA, 10 unit Tyro and 12 mM l-cys at room temperature for 45 min. Subsequently 4 time volume of pre-cold pure acetone are added and solutions are kept in −30 °C freezer 1 h before centrifuged at 13,000× *g* for 30 min to precipitate peptides. The acetone precipitation procedure is repeated to remove away any possible contaminations of tyrosinase, DA and l-cys. Finally, precipitated peptides are dissolved in 1× PBS buffer and analyzed by 12% tricine sodium dodecyl sulfate polyacrylamide gel electrophoresis (SDS-PAGE), visualized by nitroblue tetrazolium (NBT), silver staining or Coomassie Brilliant Blue R-250 (CBB R-250) dye staining protocols.

### 2.8. NBT, Silver and CBB R-250 Staining for DAQ Conjugated Peptides

DAQ conjugated peptides were detected by staining with glycinate/NBT solution (0.24 mM NBT in 2 M potassium glycinate, pH 10) [40,41]. After SDS-PAGE analysis, peptides in gels are transferred to the nitrocellulose paper. The nitrocellulose paper was immersed in the glycinate/NBT solution for 45 min in the dark resulting in a blue-purple stain of quinoprotein bands and no staining of other proteins. Then nitrocellulose was washed, photographed and/or stored in 0.1 M sodium borate, pH 10, at 4 °C. To visualize peptides in gels after SDS-PAGE analysis, gels are stained with routine CBB R-250 dye or silver nitrate according to previous published protocols [42].

### 2.9. Calcein-AM-Hoechst Fluorescent Dyes Staining of Cell Viability

The Calcein–Hoechst fluorescent dyes staining protocol was derived and modified from calcein-PI dual fluorescent cell viability detection protocol [43]. The aim to introduce Hoechst dye into the assay is to prevent any adverse influence on final fluorescent intensity due to variance of cell numbers among respective groups. In brief, 3.5 × 10^4^ cells were plated into each well of 96-well μClear tissue culture-treated black plate (Greiner Bio-One, Kremsmünster, Austria). After transfection and drugs administration, 15 μL of Opti-MEM containing Calcein-AM (1 μg/mL) and Hoechst (2 μg/mL) were added to each well. After incubation of cells at 37 °C for 30 min in the dark, the fluorescence intensity of Calcein-AM and Hoechst was measured by Tecan Infinite M200 microplate reader (GMI Inc., MN, USA) at different wavelengths: 485 nm excitation and 535 nm emission for Calcein-AM; 335 nm excitation and 460 nm emission for Hoechst. The relative fluorescent intensity of Calcein-AM was acquired via division of Calcein-AM readings with Hoechst readings of each well respectively. Cells without any treatment were set as control group. The cell viability of other groups was expressed as Cell viability (relative Calcein-AM fluorescent intensity) (% Control).

### 2.10. HPLC Analysis of DA Content

To detect DA content in DA cells and fly heads, cells and fly heads were collected and homogenized in 0.5 N perchloric acid. After centrifugation and filtration, the supernatants are used to undergo HPLC analysis subsequently. To detect DA content in solutions after oxidation, solutions are filtrated and collected for analysis after different time of reaction. The level of DA were measured using a Reversed-phase UltiMate 3000 HPLC system (Thermo Fisher Scientific, Waltham, MA, USA) with an electrochemical detector and a reversed-phase column (DBS HYPERSIL C18, 15 cm × 3.0 mm, 3 μm, 120 Å pore size, Thermo Fisher Scientific) and analyzed under the control of a Chromeleon™ 7.2 Chromatography Data System (Thermo Fisher Scientific). The mobile phase was a mixture of 0.1 M sodium phosphate, 10 mM sodium 1-heptanesulfonate, 0.1 mM EDTA and 8% methanol (*v*/*v*), adjusted to pH 2.95 with 85% phosphoric acid. All solutions for HPLC analysis were double filtered through 0.2 μm membranes and degassed before use. The flow rate was 0.5 mL per min. DA peak appears in HPLC chromatograph at about 13.5 min. DA content in solutions was acquired based on the DA peak areas in HPLC chromatography.

### 2.11. Western Blot Analysis

Cells were collected in lysis buffer (RIPA buffer (Thermo Fisher Scientific), 1% *v*/*v* protease inhibitor cocktail (Roche, Basel, Switzerland) and 2 mM phenylmethylsulfonyl fluoride (PMSF)) with the help of a cell scraper and centrifuged at 12,000× *g* at 4 °C for 20 min. From the supernatant, 30 μg proteins were resolved in 10% SDS-PAGE. The proteins were then transferred onto a PVDF, blocked in Tris buffered saline with 0.1% *v*/*v* Tween 20 (TBST) containing 5% *w*/*v* skim milk at room temperature before incubation with primary antibodies in TBST with 2% *w*/*v* skim milk (anti-GFP, Roche, Basel, Switzerland 1:1000; anti-NAD(P)H Quinone Dehydrogenase 1 (NQO-1), Santa Cruz Biotechnology, Santa Cruz, CA, USA, 1:1000; anti-Heme oxygenase 1 (HO-1), Santa Cruz Biotechnology, 1:1000; anti-PGC1-α, Novus Biologicals, Centennial, CO, USA, 1:2000; anti-β-actin, Sigma Aldrich, 1: 10,000) for overnight at 4 °C. The membrane was washed 3 x 5 min each with TBST and subsequently incubated with the secondary antibody (anti-mouse, Santa Cruz Biotechnology, CA, USA, 1:10000; anti-rabbit, Sigma Aldrich, MO, USA, 1: 5000; anti-rabbit, Sigma Aldrich, St. Louis, MO, USA, 1:10,000; anti-goat, Santa Cruz Biotechnology, 1:5000) for 1 h at room temperature. Following subsequent washes, the blots were developed with the enhanced chemiluminescent kit (Thermo Fisher Scientific) on CL-Xposure^TM^ films (Thermo Fisher Scientific).

### 2.12. Quantitative Analysis of Western Blot Data

Quantitative analysis of the densities of protein bands in western blot gels was carried out by densitometric analysis using the image software Bandscan 4.30 (BioMarin Pharmaceutical Inc., Novato, CA, USA). The ratio of the densities of respective protein bands in control lanes in Western blot gels was set as 50%. The relative densities of protein bands of other lanes were expressed as the ratio of densities, after automatic comparison with the ratio of densities of control lanes by the software.

### 2.13. Luciferase Assay

The day before transfection, HEK293T cells were cultured in 24-well plates and then transiently co-transfected with the WT and mutant ARE-pGL3-promoter vectors, WT and mutant PGC-1α promoter 2 kb luciferase vectors, mtUPR-pGL3-promoter vectors using Lipofectamine 2000 Reagent (Life Technologies, Carlsbad, CA, USA). Cells were also co-transfected with pRL-TK renilla reporter vector, which was served as internal control. Cells were harvested after 24 h post-transfection, and luciferase activity was measured with Dual-Luciferase^®^ Reporter Assay System (Promega, Wisconsin, USA) using Tecan Infinite M200 plate reader (GMI Inc., Ramsey, MN, USA). Luciferase activity was normalized to controls cells (transfected with respective vectors in the absence of any agents).

### 2.14. Drosophila Stocks, Preparation, and Behavioral Assays

Promoter lines containing ddc-GAL4 were obtained from Bloomington Drosophila Stock Center (Bloomington, IN, USA). The human A30P α-syn transgenic fly line (P{UAS-Hsap\SNCA.A30P}40.1, deposited by Prof. Nancy Bonini, University of Pennsylvania) expressing UAS controlled human mutant A30P α-syn is purchased from Bloomington Drosophila Stock Center (Bloomington, IN, USA). Yellow white control or transgenic α-syn A30P mutant flies were crossed with ddc-GAL4 lines to induce overexpression of α-syn specifically in DA neurons in transgenic fly heads. Flies were routinely raised at 25 °C on cornmeal media for 30 days in the presence or absence of 2 mM NAC, GA, TA, and l-cys before assessments of fly climbing behavior and analysis of DA content and TH positive DA neuron numbers in fly heads. For the climbing assay, motor ability was assessed at 15 min interval using the negative geotaxis assay. Briefly three cohorts of 20 female and age-matched control flies were anesthetized and placed in a vertical plastic column (length, 25 cm; diameter, 1.5 cm). After a 2 h recovery period, flies were tapped to the bottom and the percentage of flies that climb to or above the top column line in 1 min was calculated. Triplicate trials were performed in each experiment at 15 min interval.

### 2.15. MAOB Activity Assay

The MAOB assay is performed following a novel MAOB assay protocol publish recently [44]. All enzymatic assays were carried out in HEPES buffer (pH 7.5) supplemented with 0.1% Triton X-100 at 37 °C. The activity was assayed spectrophotometrically by monitoring the rate of resorufin formation at 560 nm following the manufacturer’s guidelines. All measurements were performed in triplicate. Cell lysates were prepared similarly as those for WB analysis. Next, enzymatic assays were performed in a final volume to 200 μL and 2.0 μM of the U1 probe. For inhibition assays, either rasagiline (RA) or pargyline (PA) was pre-incubated with the biological samples for 2 h before the addition of 2.0 μM U1 (a gift from Dr Zhang Chengwu and Prof Lim Kah Leong, Department of Research, National Neuroscience Institute, Singapore) for another 2–3 h. The fluorescence was monitored at 470 nm (λ_ex_ = 350 nm). The reaction constant was defined from the half-life equation for a second-order reaction dependent on one second-order reactant (k = t1/2 − 1[A]0−1). All fluorometric measurements were performed on Tecan Infinite M200 plate reader (GMI Inc.).

### 2.16. Statistical Analysis

Statistical analyses were conducted using one-way or two-way ANOVA followed by post hoc Student’s *t*-test using software Minitab v14 (Minitab, LLC, PA, USA). Graphs were constructed with Microsoft Excel 2007 (Microsoft Corporation, Washington DC, WA, USA) or SigmaPlot 2001 (Systat Software Inc., San Jose, CA, USA).

## 3. Results

### 3.1. Protection Against Metal Ions and Tyro Induced DA Oxidation by Tea Polyphenols and Other Agents

**Figure 1:**
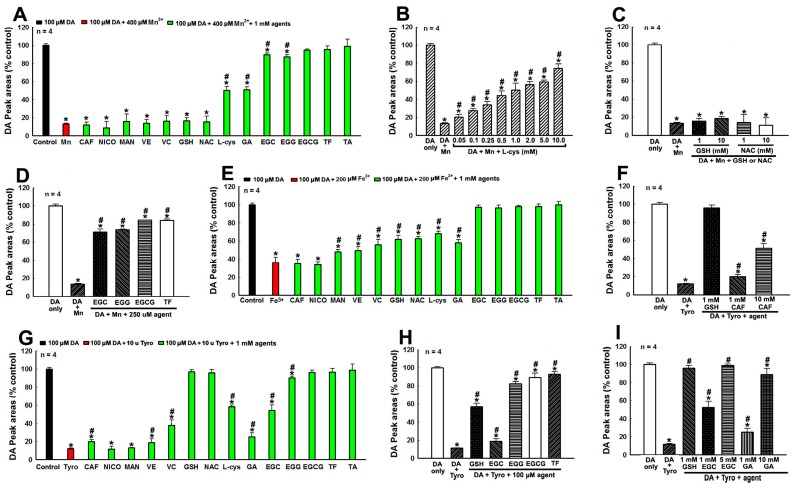
Protections against factors induced dopamine (DA) oxidation by tea polyphenols and other agents. The oxidation of 100 μM DA is induced by 400 μM Mn^3+^, 200 μM Fe^3+^ and 10-unit Tyro respectively for 3 min at room temperature in the presence or absence of various agents before HPLC analysis of DA content in solutions. Freshly prepared DA is set as control and DA peak areas are expressed as % control. *, at least *p* < 0.05, compared with DA peak areas of controls. #, at least *p* < 0.05, compared with peak areas of DA after Mn^3+^, Fe^3+^ and Tyro induced DA oxidation respectively in the absence of protective agents. (**A**–**D**), Protection against Mn^3+^ induced DA oxidation by different agents. (**A**) Protection by 1 mM agents. (**B**) Dosage dependent protection by L-cys. (**C**) glutathione (GSH) and N-acetyle-cysteine (NAC) cannot protect against Mn^3+^ induced DA oxidation. (**D**) Protection by 250 μM tea polyphenols. (**E**) Protection against Fe^3+^ induced DA oxidation by 1 mM agents. (**F**–**I**), Protection against tyrosinase induced DA oxidation by various agents. (**F**) Dosage dependent protection by CAF. (**G**) Protection by in 1 mM agents. (**H**) Protections by 100 μM tea polyphenols. (**I**) Dosage dependent protection by epigallocatechin (EGC) and GA.

Recent studies demonstrate that DA oxidation and generation of toxic by-products is highly relevant to mitochondria impairment, lysosome dysfunction, proteasome inhibition and DA neuron degeneration in PD [3,4]. Therefore, effective inhibition of DA oxidation can prevent generation of deleterious DA oxidative by-products and protect DA neurons. We investigate the capabilities of tea polyphenols and other PD-related agents to inhibit enzyme and metal ions induced DA oxidation in vitro (Figure 1). The manganese (Mn^3+^) or iron ions (Fe^3+^) are supposed to be pathological environmental factors related to PD pathogenesis [45] and we find that both of them can rapidly induce DA oxidation in solutions (Figure 1A–E). We identify that incubation of 100 μM DA with 400 μM Mn^3+^ or 200 μM Fe^3+^ at room temperature for 3 min can induce about 80% and 60% DA oxidation respectively (Figure 1A–E). The oxidation of 100 μM DA induced by 400 μM Mn^3+^ cannot be alleviated by 1 mM CAF, NICO, MAN, VE and VC as well as 1 or 10 mM GSH and NAC, but can be partially and dosage dependently inhibited by l-cys, GA, EGC, and EGG (Figure 1A–D). The 400 μM Mn^3+^ induced oxidation of 100 μM DA can be completely abrogated by 1 mM EGCG, TF and TA (Figure 1B). Furthermore, the oxidation of 100 μM DA induced by 200 μM Fe^3+^ cannot be influenced by 1 mM CAF and NICO, but can be partially inhibited by 1 mM MAN, VE, VC, GSH, NAC, L-cys, and GA (Figure 1E). The 200 μM Fe^3+^ induced oxidation of 100 μM DA can be completely abrogated by 1 mM EGC, EGG, EGCG, TF, and TA (Figure 1E). Next, we find that the oxidation of 100 μM DA induced by 10-unit Tyro can be partially abrogated by 1 mM CAF, VE, VC, l-cys, GA, and EGC, but can be completely abrogated by 1 mM GSH, NAC, EGG, EGCG, TF, and TA (Figure 1G). The inhibition of Tyro induced DA oxidation by CAF, GSH, GA, EGC, EGG, EGCG, and TF can be dosage dependent (Figure 1F,H,I). These data suggests that tea polyphenols and other agents can protect against factors induced DA oxidation with different profiles. Tea polyphenols, especially those (EGCG, TF, and TA) with more phenol or benzene rings, have excellent capabilities to abrogate various factors induced DA oxidation. Furthermore, the protective capabilities of EGCG, TF, and TA are more potent than that of GSH, the most potent and indispensable endogenous anti-oxidant and detoxification agent.

### 3.2. The Reductive Potency of Tea Polyphenols and Other Agents

**Figure 2:**
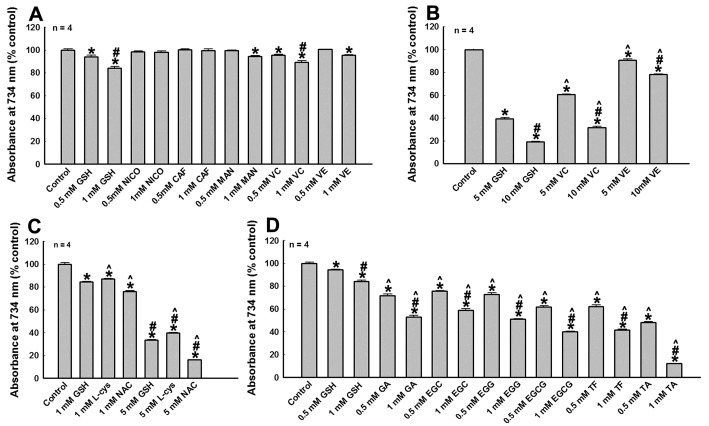
Detections of reductive potency of tea polyphenols and other agents. The reductive potencies of tea polyphenols and various Parkinson’s disease (PD)-related agents were detected by 2,2′-azino-bis(3-ethylbenzothiazoline-6-sulfonic acid) diammonium salt (ABTS) cation decolorization assay. Solutions in the absence of polyphenols and other agents are set as controls. The reductive potency of various agents at different concentration is expressed as % control of absorbance at 734 nm in ABTS cation decolorization reactions. *, at least *p* < 0.05, compared with the absorbance at 734 nm of controls. #, at least *p* < 0.05, compared with the absorbance at 734 nm of the same agents at lower concentration. ^, at least *p* < 0.05, compared with the absorbance at 734 nm of GSH at the same concentration. (**A**) Analysis of reductive potency of GSH, NICO, CAF, MAN, VC and VE at 0.5 and 1 mM dosage. (**B**) Dosage dependent reductive potency of GSH, VC and VE. (**C**) Dosage dependent reductive potency of GSH, NAC and L-cys. (**D**) Analysis of reductive potency of GSH and tea polyphenols.

Next, we study the anti-oxidative (reductive) potency of polyphenols and other agents using ABTS cation decolorization assay (Figure 2). We find that 0.5 and 1 mM NICO and CAF have no reductive potency (Figure 2A). GSH, VC, VE, and MAN have relatively weaker and dosage dependent reductive potencies (Figure 2A,B). Their reductive potencies are shown from stronger to weaker as follows: GSH ˃ VC ˃ VE ˃ MAN (Figure 2A,B). The reductive potencies of GSH, NAC, and l-cys (three agents with sulfhydryl groups) can be shown from stronger to weaker as follows: NAC ˃ GSH ˃ L-cys (Figure 2C). These results show that GSH, the significant endogenous anti-oxidative and detoxification agent, has relative stronger reductive potency. We further study and compare the reductive potency of GSH with tea polyphenols (Figure 2D). We find that the reductive potencies of all polyphenols studied are stronger than that of GSH (Figure 2D). Furthermore, polyphenols with more phenol or benzene rings have stronger reductive potency, compared with polyphenols with less phenol or benzene rings. The relative reductive forces of polyphenols and GSH are identified and shown from stronger to weaker as follows: TA ˃ TF ≈ EGCG ˃ GA ≈ EGC ≈ EGG ˃ GSH (≈ means “similar to”) (Figure 2D). Our data show that polyphenols have superior anti-oxidative and reductive potencies, compared with other agents studied.

### 3.3. MAOB Inhibition Capabilities of tea Polyphenols and Other Agents

**Figure 3:**
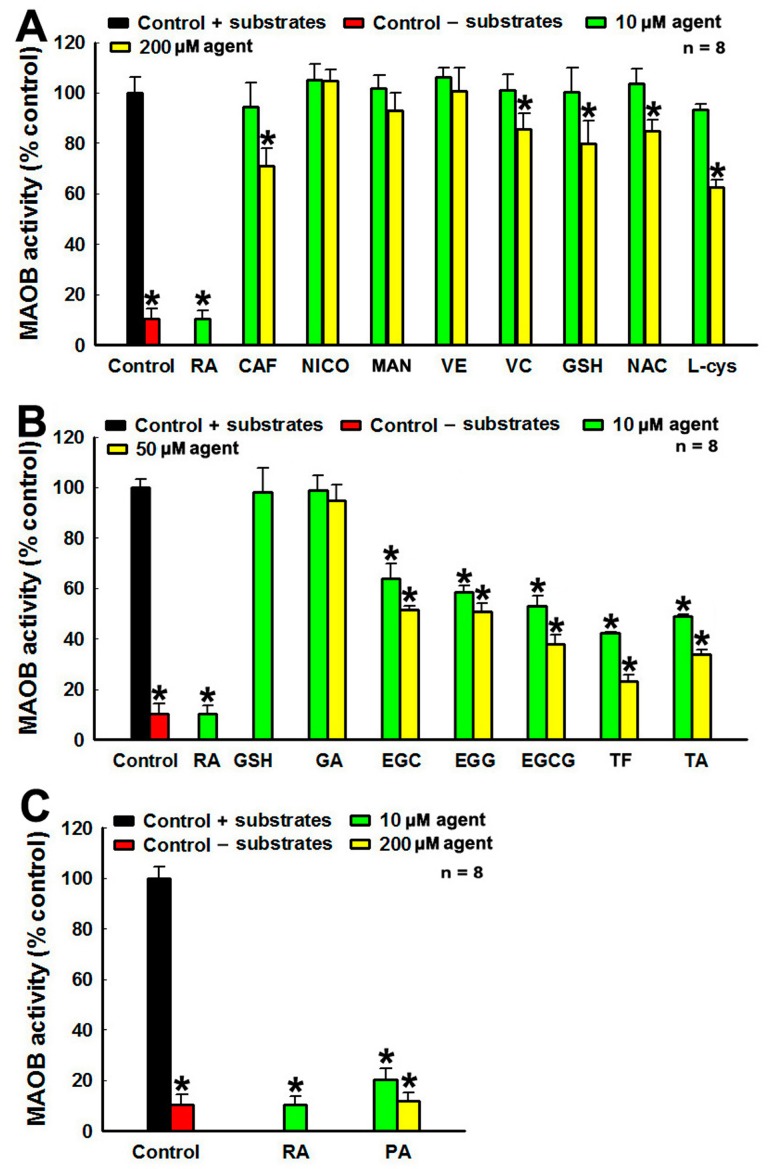
Inhibitions of monoamine oxidase B (MAOB) activity by tea polyphenols and other agents. MAOB activities of human dopaminergic SH-SY5Y cell lysates in the presence of MAOB inhibitors and tea polyphenols as well as other agents are detected. MAOB activity of cell lysates in the absence of MAOB inhibitor and other agents is set as controls. MAOB activity of cell lysates in the presence of MABO inhibitors or other agents is expressed as % control. *, at least *p* < 0.05, compared with MAOB activity of controls. (**A**) MAOB inhibition by 10 and 200 μM caffeine (CAF), nicotine (NICO), mannitol (MAN), α-Tocopherol (VE), ascorbic acid (VC), GSH, NAC, and L-cys. (**B**) MAOB inhibition by 10 and 50 μM tea polyphenols. (**C**) rasagiline (RA) and pargyline (PA) induced MAOB inhibition.

The mitochondria localized MAOB can catalyze DA degradation, leading to ROS generation, which is implicated in the PD pathogenesis [46]. Therefore, we examine the potential capabilities of polyphenols and other agents to inhibit MAOB activity of human dopaminergic SH-SY5Y cells with a recently developed MAOB assay [44]. We find that 10 μM RA or 200 μM PA, two specific MAOB inhibitors, can significantly inhibit MAOB activity of SH-SY5Y cells in vitro (Figure 3C). Incubation of SH-SY5Y cell lysate with 10 μM PA can lead to abrogation of 90% MAOB activity (Figure 3C). No change in MAOB activities was observed in the lysate incubated with 10 and 200 μM NICO, MAN, and VE (Figure 3A). The MAOB activity of SH-SY5Y cells can be slightly down regulated by 200 μM CAF, VC, GSH, NAC, and l-cys (Figure 3A). Furthermore, GA with mono-phenol ring has no significant influence on MAOB activity (Figure 3B). However, polyphenols with multiple ring structures, including EGC, EGG, EGCG, TF, and TA, show significant and dosage dependent inhibition of MAOB activity (Figure 3B). Incubation of cell lysate with 50 μM TF can abrogate about 80% MAOB activity of SH-SY5Y cells. Our data show that polyphenols show considerable potency to inhibit MAOB activity of human dopaminergic cells.

### 3.4. Modulation of Anti-Oxidative and Proliferative Nrf2-Keap1 and PGC1α Signaling Pathways by Tea Polyphenols and Other Agents

**Figure 4:**
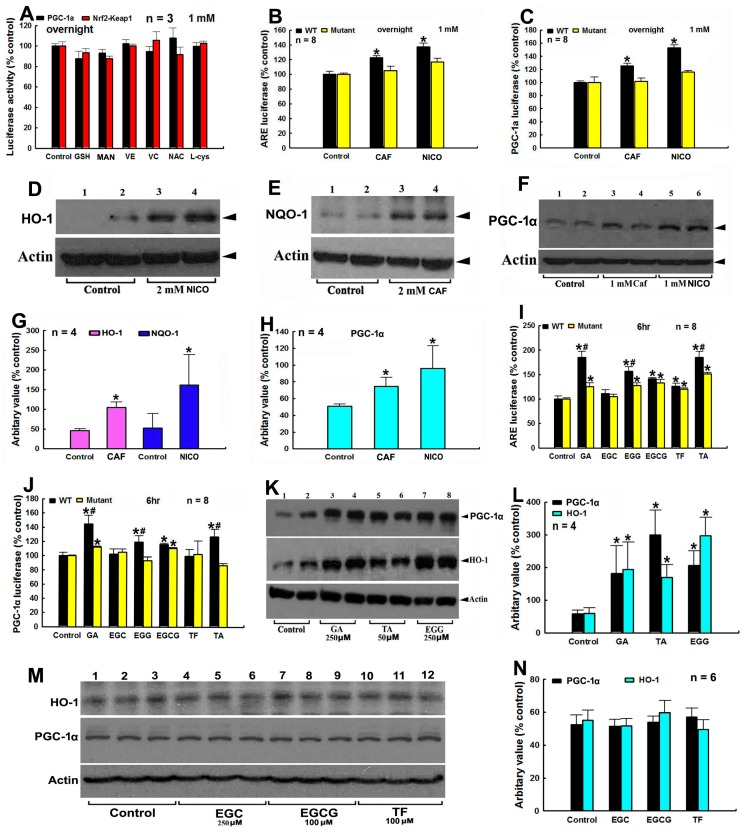
Modulations of Nrf2-Keap1 and PGC-1α signaling pathways by tea polyphenols and other agents. (**A**–**C**), potential modulations of nuclear factor erythroid 2-related factor 2 (Nrf2)-Keap1 and Peroxisome proliferator-activated receptor gamma coactivator 1-alpha (PGC-1α) signaling pathways by tea polyphenols and other agents are monitored in antioxidant response element (ARE)-Luciferase and PGC-1α promoter luciferase vectors transfected HEK cells respectively after overnight treatment by various agents. Cells were lyzed and luciferase activities are analyzed. (**A**–**C**), monitoring of agents induced modulation of Nrf2-keap1 or PGC-1α signaling pathways by luciferase assay. *, *p* < 0.001, compared with the luciferase value of control cells. (**A**) Modulation of Nrf2-keap1 pathway by GSH, Mann, VE, VC, NAC, and l-cys. (**B**) Modulation Nrf2-keap1 pathway by NICO and CAF. (**C**) Monitoring of NICO and CAF induced modulation of PGC-1α pathway. (**D**–**H**) Agents induced influence on Nrf2-Keap1 and PGC-1α signaling pathways in SH-SY5Y dopaminergic cells. (**D**–**F**) Representative western blot gel picture of HO-1, NQO-1, and PGC-1α protein bands in the presence or absence of NICO or CAF. (**D**), HO-1 protein bands under NICO treatment; (**E**) NQO-1 protein bands under CAF treatment; (**F**), PGC-1α protein bands under CAF and NICO treatments. (**G**,**H**) Quantitative analysis of NICO or CAF induced up-regulated expressions of HO-1 and NQO-1 (**G**) and PGC-1α (**H**), based on densitometric scanning of protein bands in Western blot gels. *, at least *p* < 0.05, compared with the densitometric value of protein bands of cells without NICO or CAF treatments. (**I**,**J**), monitoring of 6 hr polyphenols treatment induced modulation of Nrf2-keap1 (**I**) or PGC-1α (**J**) signaling pathways by luciferase assay. *, at least *p* < 0.05, compared with the luciferase value of control cells. #, at least *p* < 0.01, compared with the luciferase value of cells transfected with mutant luciferase vectors (**K**,**L**), polyphenols induced modulations of Nrf2-Keap1 and PGC-1α signaling pathways in SH-SY5Y dopaminergic cells, validated by Western blot analysis. (**K**) Representative western blot gel picture of HO-1 and PGC-1α protein bands after 6 h treatments by GA, EGG and TA, (**L**) quantitative analysis data based on densitometric scanning of HO-1 and PGC-1α protein bands in western blot gels. *, at least *p* < 0.05, compared with the respective densitometric value of HO-1 and PGC-1α protein bands of cells without polyphenols treatments. (**M**) and (**N**), EGC, EGCG, and TF fail to modulate Nrf2-Keap1 and PGC-1α signaling pathways in SH-SY5Y dopaminergic cells. (**M**) Representative western blot gel picture of HO-1 and PGC-1α protein bands after 6 hr treatments by EGC, EGC, and TF, (**N**), quantitative analysis data based on densitometric scanning of HO-1 and PGC-1α protein bands in western blot gels.

Subsequently we investigate the roles of polyphenols and other agents in modulations of anti-oxidative and proliferative signaling pathways, including Nrf2-Keap1 and PGC1α signaling pathways. To monitor agents induced modulations of Nrf2-Keap1 signaling pathway, specific ARE controlled luciferase vectors (WT and mutant ARE-pGL3 promoter plasmids) are constructed and utilized (Supplementary Figure S3). WT and mutant PGC1α promoter controlled pGL3 luciferase vectors are purchased from Addgene. Based on data achieved from luciferase assay, we find that overnight treatments of cells with GSH, MAN, VE, VC, NAC, and l-cys have no influence on Nrf2-Keap1 and PGC1α signaling pathways (Figure 4A). However, overnight treatment of cells with CAF and NICO, especially NICO, can significantly enhance luciferase signals in WT ARE and WT PGC-1α promoter-controlled luciferase vectors transfected cells, but not in mutant ARE and mutant PGC-1α promoter controlled luciferase vectors transfected cells (Figure 4B,C). Furthermore, NICO is more potent than CAF to positively modulate Nrf2-Keap1 and PGC1α pathways (Figure 4B–H). The positive modulations of Nrf2-Keap1 and PGC1α signaling pathways by CAF and NICO is further validated by Western blot analysis of HO-1, NQO-1, and PGC-1α protein levels in CAF and NICO treated SH-SY5Y cells (Figure 4D,H).

We further find that that treatment of cells with GA, EGG, and TA for 6 hrs can significantly increase luciferase signals of cells transfected with WT ARE-pGL3 and WT PGC-1α promoter luciferase vectors, compared with luciferase signals of cells transfected with mutant ARE-pGL3 and mutant PGC-1α pGL3 luciferase vectors (Figure 4I,J). Western blot analysis show up-regulated expressions of PGC-1α and HO-1 proteins in SH-SY5Y cells after GA, EGG and TA 6 hr treatments, suggesting that GA, EGG and TA are positive modulators of Nrf2-Keap1 and PGC1α signaling pathways (Figure 4K,L). However, EGC, EGCG and TF cannot significantly enhance luciferase signals of cells transfected with WT ARE-pGL3 and WT PGC-1α promoter luciferase vectors, compared with cells transfected with mutant ARE-pGL3 and mutant PGC-1α promoter luciferase vectors (Figure 4I,J). Western blot analysis also confirms that EGC, EGCG, and TF cannot up-regulate the levels of PGC-1α and HO-1 proteins in SH-SY5Y cells (Figure 4M,N). These findings demonstrate that not all polyphenols can activate Nrf2-Keap1 and PGC1α anti-oxidative and proliferative signaling pathways.

### 3.5. Protection against DA Relevant PC12 Cell Death by Tea Polyphenols and Other Agents

**Figure 5:**
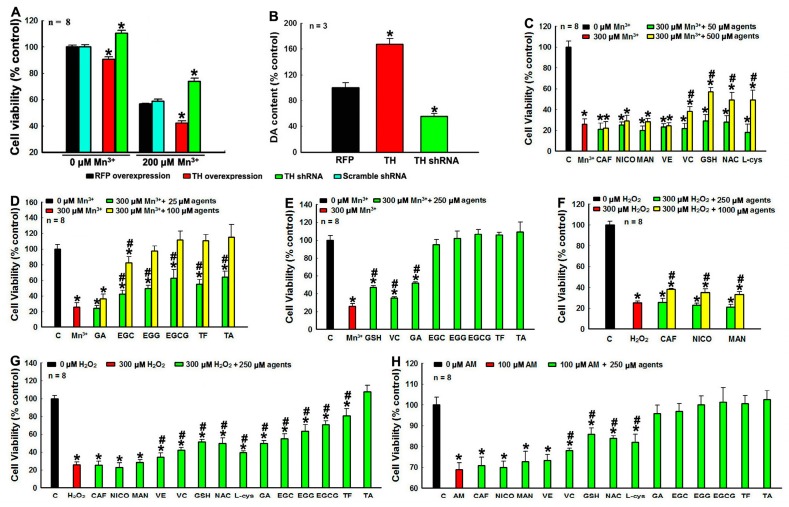
Protection against Mn3+, H2O2, and AM induced dopaminergic PC12 cell death by tea polyphenols and other agents. Dopaminergic PC12 cells were challenges with 200 or 300 μM Mn^3+^ or 300 μM H_2_O_2_ overnight or 100 μM AM 3 h respectively in the presence or absence of various agents. Cells without any challenges are set as control. *, at least *p* < 0.05, compared with cell viability of control cells. #, at least *p* < 0.05, compared with cell viability of cells challenged with stressors only. (**A**,**B**), The Mn^3+^ induced cell toxicity are dependent on endogenous DA level in PC12 cells. (**A**) Influence on PC12 cell viability by tyrosine hydroxylase (TH) overexpression or knockdown under Mn^3+^ overnight challenge. PC12 cells were transfected with rat-TH or TH shRNA vectors overnight respectively, before subsequent 200 μM Mn^3+^ overnight challenge. (**B**) Influence on DA level in PC12 cells by TH overexpression or knockdown. (**C**–**E**) Protection of PC12 cells against 300 μM Mn^3+^ overnight challenges induced toxicity by different agents. (**C**) Protection by 50 and 500 μM CAF, NICO, MAN, VE, VC, GSH, and l-cys. (**D**) Protection by 25 and 100 μM GA, EGC, EGCG, TF, and TA. (**E**) Protection by 250 μM GSH, l-cys and tea polyphenols. (**F**–**H**), protection against 300 μM H_2_O_2_ or 100 μM AM challenges induced toxicity by various agents. (**F**) Protection against H_2_O_2_ induced toxicity by 250 and 1000 μM CAF, NICO, and MAN. (**G**) Protection against H_2_O_2_ induced toxicity by 250 μM agents. (**H**) Protection against 100 μM AM induced toxicity by 250 μM agents.

We find that CAF, NICO, MAN, GSH, NAC, and L-cys have no toxic effects on PC12 cell (Supplementary Figure S2A–F). Treatments with EGCG and TF less than 500 μM have no deleterious impacts on of PC12 cell viability (Supplementary Figure S2G,H). To check polyphenols and other agents induced protection to DA neurons, Mn^3+^ ion is selected as an environmental stressor, as it is highly relevant to PD with strong potency to mediate DA oxidation to induce DA cell toxicity [47,48]. Our current findings show that Mn^3+^ ion can rapidly and effectively mediate DA oxidation in solutions (Figure 1A). Furthermore, we find that the toxicity of Mn^3+^ ion is dependent on endogenous DA levels in dopaminergic PC 12 cells (Figure 5A,B). Overexpression of TH in PC12 cells can significantly up-regulate DA level, leading to increased sensitivity of PC12 cells to 200 μM Mn^3+^ ion induced challenge (Figure 5A,B). However, knockdown of endogenous TH decrease DA levels, which significantly alleviate 200 μM Mn^3+^ ion induced PC12 cell death (Figure 5A,B). These findings suggest that Mn^3+^ ion induce oxidation of endogenous DA in PC12 cells, which significantly account for Mn^3+^ ion induced PC12 cell demise. We further find that the challenge of PC12 cell with 300 μM Mn^3+^ can lead to 70% cell death of PC12 cells (Figure 5C–E). Furthermore, PC12 cell death induced by 300 μM Mn^3+^ challenge cannot be rescued by 50 μM or 500 μM CAF, NICO, MAN and VE, but can be partially rescued by 500 μM VC, GSH, NAC and l-cys (Figure 5C). We show that 300 μM Mn^3+^ challenge induced dopaminergic PC12 cell demise can be significantly and dosage dependently protected by polyphenols (Figure 5D,E). The PC12 cell death induced by 300 μM Mn^3+^ challenge can be completely abrogated by 100 μM EGG, EGCG, TF and TA and 250 μM EGC (Figure 5D,E). These findings implicate that tea polyphenols function better than other agents to protect against DA-related toxicity in DA cells.

The excellent protective capabilities of tea polyphenols against DA-related toxicity are further demonstrated by tea polyphenols induced protections against DA oxidative by-products (ROS and DAQ) induced toxicity. We find that polyphenols can significantly protect PC12 cells against H_2_O_2_, one kind of DA oxidative ROS by-product, induced toxicity (Figure 5F,G). The PC12 cell death induced by 3 h 300 μM H_2_O_2_ challenge can only be partially rescued by higher dosage (1000 μM) of CAF, NICO, and MAN (Figure 5F). However, PC12 cell death induced by 300 μM H_2_O_2_ challenge can be completely rescued by 250 μM TA, but partially rescued by 250 μM VE, VC, GSH, NAC, L-cys, GA, EGC, EGG, EGCG, and TF to different extents (Figure 5G). Compared with other agents, polyphenols with more ring structures demonstrate better protections against H_2_O_2_ induced PC12 cell death (Figure 5G). This result is conformed to our finding that reductive potency of tea polyphenols is in proportion to the numbers of ring structure of polyphenols (Figure 2D).

The oxidation of DA can generate small ROS as well as highly reactive DAQ. ROS can induce reversible oxidative modification, while DAQ will irreversibly conjugate with protein residues to induce protein misfolding, inactivation and aggregation. Therefore, we further assess the protective effects of tea polyphenols and other agents against AM (a cyclized DAQ generated from DA oxidation) induced PC12 cell death. We find that PC12 cell death induced by 100 μM AM 3 hr challenge cannot be rescued by 250 μM CAF, NICO, MAN, or VE, but can be partially rescued by 250 μM VC, GSH, NAC, and L-cys (Figure 5H). This is conformed to our previous findings that GSH and other agents with sulfhydryl groups can competitively react with DAQ to protect proteins in DA cells [6,7]. However, PC12 cell death induced by 100 μM AM challenge can be completely abrogated by 250 μM polyphenols, including GA, EGC, EGG, EGCG, TF, and TA (Figure 5H). Our findings demonstrate for the first time that tea polyphenols with hydroxyl groups can detoxify DAQ and protect DA cells. Furthermore, our data implicates that the potency of polyphenols to conjugate with DAQ is stronger than that of GSH, the key endogenous DAQ quencher. The MAN also has six hydroxyl groups; however, MAN does not have any protection against AM induced PC12 cell toxicity (Figure 5H). Therefore, our data implicates that phenolic hydroxyl groups in ring structures is specific to react with DAQ, whereas hydroxyl groups in straight carbon chain do not have such kind of function. Taken together, our findings demonstrate that, compared with other agents, polyphenols demonstrate excellent protections against DA-related toxicity in DA cells.

### 3.6. Phenolic Hydroxyl Groups Can React with DAQ

**Figure 6:**
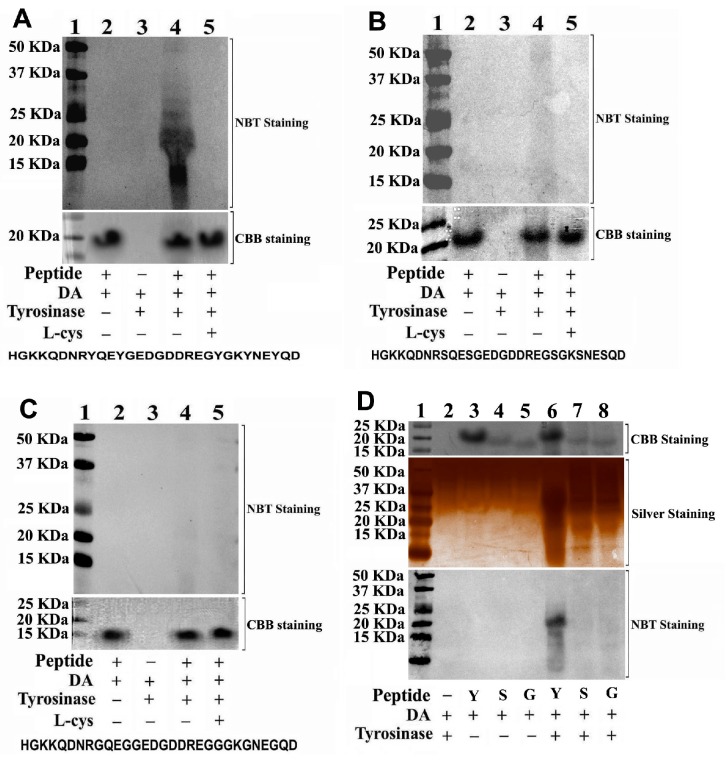
Conjugations of DA quinones (DAQ) to phenolic hydroxyl groups of synthesized peptides. Three peptides synthesized react with DA under tyrosinase catalysis in the presence or absence of l-cys. After reaction, peptides are precipitated and analyzed by SDS-PAGE, visualized by nitroblue tetrazolium (NBT), silver, and CBB R-250 staining respectively. (**A**) Peptide Y with 5 phenolic hydroxyl groups (tyrosine residues) can react with DAQ well in solutions, (**B**) peptide S with 5 non-phenolic hydroxyl groups (serine residues) have poor capability to react with DAQ. (**C**) Peptides G with no hydroxyl groups (glycine residues) have poor capability to react with DAQ in solutions. (**D**) Comparison of reactions capability of 3 peptides with DAQ.

To further verify whether phenolic hydroxyl groups can react and conjugate with DAQ or not, we design and synthesize 3 peptide fragments with 30 amino acids (Supplementary Figure S4). The peptide G does not have any sulfhydryl or hydroxyl groups. The peptide S has 5 serine residues; therefore, it contains 5 non-phenolic hydroxyl groups in branch straight carbon chain of the peptide. The peptide Y has 5 tyrosine residues; therefore, it has 5 phenolic hydroxyl groups. We find that 1.5 mM peptide Y can react with DAQ generated from oxidation of 6 mM DA, which can be abrogated by 12 mM l-cys (Figure 6A). In the absence of 10 u Tyro, no conjugations between peptide Y and DAQ can be found, confirming DAQ conjugation with peptide Y after Tyro catalyzed DA oxidation (Figure 6A). However, we find that peptide S and peptide G have very poor capabilities to conjugate with DAQ (Figure 6B–D). The significant reaction capability of peptide Y with DAQ is further compared and visualized in Figure 6D. These findings demonstrate the active reaction of phenolic hydroxyl groups with DAQ for the first time, supporting the direct conjugations of phenolic hydroxyl groups of polyphenols with DAQ to abrogate DAQ induced conjugations of functional proteins. Our findings confirm that the phenolic hydroxyl groups can react with DAQ, whereas non-phenolic hydroxyl groups in straight carbon chain do not have this function. Furthermore, our findings implicated that, beside sulfhydryl groups of proteins, phenolic hydroxyl groups of proteins can also conjugate with DAQ, leading to DAQ induced protein misfolding and inactivation.

### 3.7. Protection against Overexpression of Mutant A30P α-Syn Induced DA Neuron Degeneration in Transgenic Fly Heads by Tea Polyphenols and Other Agents

**Figure 7:**
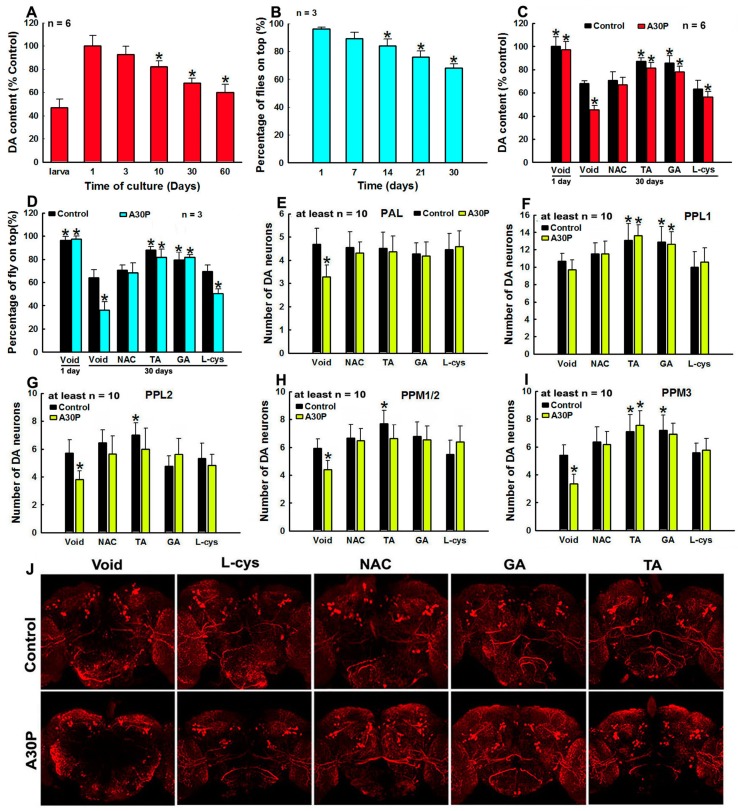
Protection against overexpression of mutant A30P α-synuclein (α-syn) induced DA neuron degeneration in fly head by GA, TA, NAC, and l-cys. Yellow white control or transgenic α-syn A30P mutant flies were crossed with ddc-GAL4 lines to induce overexpression of A30P mutant α-syn specifically in DA neurons in transgenic fly heads. Flies were cultured for 30 days in the presence or absence of 2 mM GA, TA, NAC, and l-cys before check of fly climbing behavior and analysis of DA content and TH positive DA neuron numbers in fly heads. Flies without any drug treatments are set as void group. *, at least *p* < 0.05, compared with yellow white void flies without any drug treatment. (**A**,**B**), age dependent decrease of DA contents (**A**) and impairment of climbing capabilities (**B**) of yellow white files; (**C**) HPLC analysis of DA contents in transgenic fly heads; (**F**), monitoring climbing capabilities of transgenic flies. (**E**–**I**), numbers of TH positive DA neurons in different districts of transgenic fly heads. (**E**), PAL; (**F**), PPL1; (**G**), PPL2; (**H**), PPM1/2; (**I**), PPM3. (**J**) Confocal fluorescent images of TH positive DA neurons in transgenic fly heads with or without treatments by various agents.

We further investigate agents induced protections against overexpression of human mutant A30P α-syn induced DA neuron degeneration in transgenic fly heads (Figure 7). Among various agents studied, in vitro potent protective agents including NAC, l-cys, GA (the smallest polyphenol) and TA (the biggest polyphenol) are selected to be tested in our in vivo transgenic PD model. We notice that culturing of drosophila with time in the absence of A30P α-syn demonstrates ageing dependent decline of DA content in fly heads and impaired climbing capabilities, suggesting natural ageing relevant vulnerability of DA neurons in fly heads (Figure 7A,B). However, overexpression of human mutant A30P α-syn in DA neurons in fly heads for 30 days can induce significant fly heads DA neuron degeneration, validated with significantly impaired climbing capability, decreased DA content and DA neuron loss in fly heads (Figure 7C–J). However, the DA neuron degeneration induced by overexpression of human mutant A30P α-syn can be rescued by NAC, l-cys, GA and TA to different extents (Figure 7C–J). NAC treatment can completely abrogate overexpression of mutant A30P α-syn induced DA neuron degeneration in fly heads (Figure 7C–J). The protective capability of l-cys to protect fly DA neurons against mutant A30P α-syn induced toxicity is slightly weaker than that of NAC (Figure 7C–J). However, polyphenols TA and GA, especially TA, have the most potent protective effects on fly head DA neurons (Figure 7C–J). Both TA and GA treatments can completely abrogate overexpression of mutant A30P α-syn induced DA neuron degeneration in fly heads (Figure 7C–J). Furthermore in the presence of TA and GA, fly climbing capability, DA contents in fly heads and numbers of DA neurons in fly heads of flies with overexpression of mutant A30P α-syn can be significantly higher than those of yellow-white control flies in the absence of mutant A30P α-syn and polyphenols (Figure 7C,D). These findings implicate that TA and GA can counteract against mutant A30P α-syn as well as natural ageing process induced deleterious impacts on DA neurons in fly head. Based on our achieved findings, the protective potency of agents on fly head DA neurons can be demonstrated from stronger to weaker as follows: TA ˃ GA ˃ NAC ˃ l-cys.

## 4. Discussion

PD is an incurable human neurodegenerative disease and no drugs or therapies have been developed to alleviate the progressive DA neuron degeneration in PD brains so far. The pathogenesis of PD is still under debate. Recent findings highlight that oxidation of endogenous DA can lead to DA-related toxicity including mitochondria impairment, proteasome inhibition, lysosome dysfunction, and selective DA neuron vulnerability [3,4,5,6,49]. The DA can undergo auto-oxidation or oxidation induced by metal ions (Mn or Fe) or specific enzymes (Tyro or MAO). DA oxidation generates deleterious ROS and highly reactive DAQ, which can contribute to DA neuron vulnerability. Therefore, strategies to abrogate DA oxidation by ion chelation, MAOB inhibition and reductive force enhancement as well as agents to diminish ROS by ROS scavengers and to detoxify DAQ by DAQ quenchers can protect DA neurons against DA-related toxicity. Accumulative evidence suggests that smoking and drinking of coffee or tea are associated with lower risk of PD with unclear mechanisms. In this study we discover that tea polyphenols are more potent and versatile to protect against various pathological factors induced DA neuron degeneration in PD models than other agents. We identify that tea polyphenols can effectively protect DA neurons against DA-related toxicity. We find that tea polyphenols can abrogate DA oxidation, scavenge ROS, conjugate and detoxify DAQ, inhibit MAOB and modulate anti-oxidative proliferative signaling pathways, which is significant to tea polyphenols induced protection of DA neurons. Furthermore, we uncover that the capabilities of tea polyphenols to protect DA neurons are dependent on the numbers of ring structures and amounts of phenolic hydroxyl groups of tea polyphenols (Table 1). Tea polyphenols with more ring structures and phenolic hydroxyl groups are more potent to protect DA neurons (Table 1). The polyphenols have been supposed to have therapeutic implications in multiple neurological disorders including depression, ischemia/reperfusion injury, neuroinflammation, glutamate-induced neuron toxicity, epilepsy, traumatic brain injury as well as hearing and vision disturbances [50]. Furthermore, polyphenols are identified to be neuroprotective for multiple human neurodegenerative diseases including PD, Huntington’s disease (HD) and amyotrophic lateral sclerosis (ALS) [50]. It was reported that polyphenols, including GA, propyl gallate (PG), epicatechin (EC), EGC, and EGCG, can protect DA neurons and restore the impaired movement activity induced by iron and/or paraquat (PQ) challenges in Drosophila PD model [51,52]. Furthermore, green and black tea polyphenol extracts, as well as EGCG, were identified to be able to protect against MPTP or 6-OHDA challenges induced DA neurons degeneration in mice, rats and monkey PD models [53,54]. The EGCG and other epigallocatechin from green tea have been reported to cross through blood–brain barrier (BBB) in animal models efficiently, regardless of their route of administration [55,56]. Currently EGCG has reached phase II clinical trials for anti-PD drug development [36]. Therefore, our findings further support that tea polyphenols can be promising drug candidates with potent and versatile protective capabilities, which will add to our anti-PD drug developments.

Previous evidence showed that NICO and CAF are negatively related to PD risk and have DA neuron protective potency [25,57]. The NICO and CAF were supposed to protect DA neurons via multiple mechanisms [28,29,30,31,32,33,34,35]. We discovered for the first time that both of them, especially NICO, can significantly activate Nrf2-keap1 and PGC-1α anti-oxidative signaling pathways. The protective Nrf2-keap1 and PGC-1α signaling pathways function to maintain the redox homeostasis and promote mitochondria biogenesis and cell proliferation, which has been potentially linked to PD pathogenesis and therapy [17,58]. However, neither NICO nor CAF has strong inhibiting capabilities on MAOB activity. The NICO and CAF do not have any ROS scavenge and DAQ quenching activities. Neither NICO nor CAF has strong potency to abrogate factors induced DA oxidation. In the current study it is shown that at higher dosage NICO and CAF have weak protective capabilities against H_2_O_2_ induced PC12 cell death. However, neither NCIO nor CAF can protect against Mn^3+^ or AM challenges induced PC12 cell death. Taken together, our findings suggest that activation of protective Nrf2-Keap1 and PGC-1α proliferative signaling pathways should account for NICO and CAF induced protection of DA cells, which is supposed to be relevant to the reduced PD risk by smoking and drinking of coffee.

In this study we also validate the DA neuron protective capabilities of GSH, NAC and l-cys, three sulfhydryl group containing protective agents. Previous findings show that GSH can protect DA neurons via suppression of DA-related toxicity including inhibition of DA oxidation, covalently conjugation with DAQ and scavenge of ROS [6,7,59]. In the current study we show the stronger protective capabilities of GSH, NAC and l-cys than those of NICO, CAF, VE VC, and MAN. Additionally, GSH, NAC and L-cys are identified to have different protective profiles respectively. The anti-oxidative potency of GSH, NAC and l-cys can be ranged from stronger to weaker as follow: NAC ˃ GSH ˃ l-cys. Both GSH and NAC are stronger than l-cys to inhibit Tyro catalyzed DA oxidation. However, l-cys is stronger than GSH and NAC to inhibit MAOB enzyme activity. The l-cys can abrogate Mn^3+^ induced DA oxidation dosage dependently, whereas GSH and NAC are unable to do so, even at their higher dosages. In our PD models, we find that GSH and NAC are more potent than l-cys to protect against stresses induced PC12 cell death. The NAC is also found to be stronger than l-cys to protect against transgenic mutant A30P α-syn induced fly DA neurons degeneration. The GSH is one of the most abundant endogenous antioxidant and detoxifying agent in brain, which plays an important role in the protection of neurons via detoxifying deleterious oxidative by-products [60]. Postmortem studies demonstrated that there was an obvious decrease (~50%) of GSH level in SN of PD patients compared with aged controls, [61]. The GSH level is not reduced in other areas of PD brains or in other diseases affecting the basal ganglia, such as multiple system atrophy (MSA) or progressive supranuclear palsy (SP) [62]. Furthermore, the depletion of GSH in DA neurons can lead to selective inhibition of mitochondrial complex I, followed by DA neurodegeneration in mice PD model [60]. Therefore, GSH is highly relevant to PD pathogenesis [63]. Similar to GSH, NAC and l-cys also contain sulfhydryl groups and they can be the precursors of GSH [35]. NAC was found to abrogate 6-hydroxydopamine (6-OHDA) induced cell death in cultured neuronal cells [64]. NAC was also found to improve mitochondrial activities of aged mice [65]. NAC and l-cys can protect against overexpression of mutant A53T α-syn induced PC12 cell death [66]. Furthermore, clinical trial administrations of NAC to PD patients have significantly improved the Unified Parkinson’s Disease Rating Scale (UPDRS) scores of PD patients [8]. So far NAC is proposed to be a promising anti-PD drug [8]. However, we identify that GSH, NAC and L-cys are unable to modulate protective Nrf2-Keap1 and PGC-1α signaling pathways. Furthermore, the capabilities of GSH, NAC and l-cys to inhibit DA oxidation, scavenge ROS, inhibit MAOB, conjugate and detoxify DAQ as well as protect against DA neuron degeneration in our PD models are inferior to those of tea polyphenols. Therefore, the potential contributions of tea polyphenols and their derivatives to future anti-PD drugs development should be highlighted based on findings from our current study.

In the current study, we demonstrate that tea polyphenols can significantly protect against both environmental and genetic factors induced DA cell degeneration in our in vitro and in vivo PD models. We identify that tea polyphenols can significantly suppress DA-related toxicity including abrogation of DA oxidation, inhibition of MAOB, detoxifications of both ROS and DAQ, two classes of toxic DA oxidative by-products and modulations of anti-oxidative Nrf2-Keap1 and PGC-1α pathways. Furthermore, our data implicate that the protective capabilities of tea polyphenols against DA-related toxicity are dependent on and related to the numbers of ring structures and amounts of phenolic hydroxyl groups of tea polyphenols. We highlight the implications of DA-related toxicity in DA neuron degeneration of PD pathogenesis, whereas tea polyphenols are supposed to protect against DA-related toxicity via direct abrogation of DA oxidative by-products induced toxicities as well as modulations of anti-oxidative signaling pathways.

## Figures and Tables

**Table 1:** Structure features and identified functions of tea polyphenols and other agents.

Chemicals	Numbers of Ring Structures	Numbers of Hydroxyl Groups	Numbers of Sulfhydryl Groups	Reductive Potency at 1 mM Dosage (% ABTS Reduced)	MAOB inhibiting (% Inhibited)	DAQ Detoxification Capabilities	Nrf2-Keap1 Pathway Modulation	PGC-1α Pathway Modulation
GSH	0	0	1	16.0	0.0	+	−	−
NAC	0	0	1	24.0	0.0	+	−	−
L-cys	0	0	1	13.0	7.6	+	−	−
CAF	0	0	0	0.0	6.6	−	+	+
NICO	0	0	0	0.0	0.0	−	+	+
VC	1	2	0	10.0	0.0	+	−	−
VE	2	1	0	4.7	0.0	−	−	−
MAN	0	6	0	5.6	0.0	−	−	−
GA	1	3	0	47.0	0.0	+	−	−
EGC	3	6	0	42.0	36.2	+	+	+
EGG	4	7	0	49.0	41.6	+	+	+
EGCG	4	8	0	69.0	47.0	+	−	−
TF	6	9	0	59.0	57.7	+	−	−
TA	10	25	0	88.0	51.2	+	+	+

## Supplementary Materials

The following are available online at https://www.mdpi.com/2073-4409/8/8/911/s1, Figure S1: Molecular structures of tea polyphenols and other agents, Figure S2: Impacts of various agents on cell viability of PC12 cells, Figure S3: Vector constructive map of ARE-pGL3promoter vector, Figure S4: HPLC and LC-MS-MS analysis of 3 peptides synthesized.

## Abbreviations:

6-OHDA	6-hydroxydopamine
α-syn	α-synuclein
ABTS	2,2′-azino-bis(3-ethylbenzothiazoline-6-sulfonic acid) diammonium salt
ALS	amyotrophic lateral sclerosis
AM	aminochrome
ARE	antioxidant response element
BBB	blood brain barrier
CAF	caffeine
CBB R-250	Coomassie Brilliant Blue R-250
DA	dopamine
DAQ	dopamine quinone
EC	epicatechin
EGC	epigallocatechin
EGCG	epigallocatechin-3-gallate
EGG	epicatechin-3-gallate
GA	Garlic acid
GSH	glutathione
H_2_O_2_	hydrogen peroxide
HD	Huntington’s disease
HO-1	Heme oxygenase 1
L-cys	L-cysteine
MAN	mannitol
MAOA	monoamine oxidase A
MAOB	monoamine oxidase B
MSA	multiple system atrophy
NAC	N-acetyl-cysteine
nAChRs	nicotinic acetylcholine receptors
NICO	nicotine
NBT	nitroblue tetrazolium
NQO-1	NAD(P)H Quinone Dehydrogenase 1
NO	nitric oxide
Nrf2-Keap1	nuclear factor erythroid 2-related factor 2-Keap1
PA	pargyline
PD	Parkinson’s disease
PG	propyl gallate
PGC-1α	Peroxisome proliferator-activated receptor gamma coactivator 1-alpha
PMSF	phenylmethylsulfonyl fluoride
PQ	paraquat
RA	rasagiline
ROS	reactive oxygen species
SDS-PAGE	sodium dodecyl sulfate polyacrylamide gel electrophoresis
SN	substantia nigra
SP	progressive supranuclear palsy
TA	tannic acid
TF	theaflavins
TH	tyrosine hydroxylase
Tyro	tyrosinase
VC	ascorbic acid
VE	α-Tocopherol
WT	wild type
