# Supplementary material for: The Therapeutic Implications of Tea Polyphenols against Dopamine (DA) Neuron Degeneration in Parkinson’s Disease (PD)
Source: Cells. 2019 Aug 16;8(8):911. doi: 10.3390/cells8080911 (PMC6721683; doi:10.3390/cells8080911)
Supplement: Supplementary file 1 [file cells-08-00911-s001.pdf]

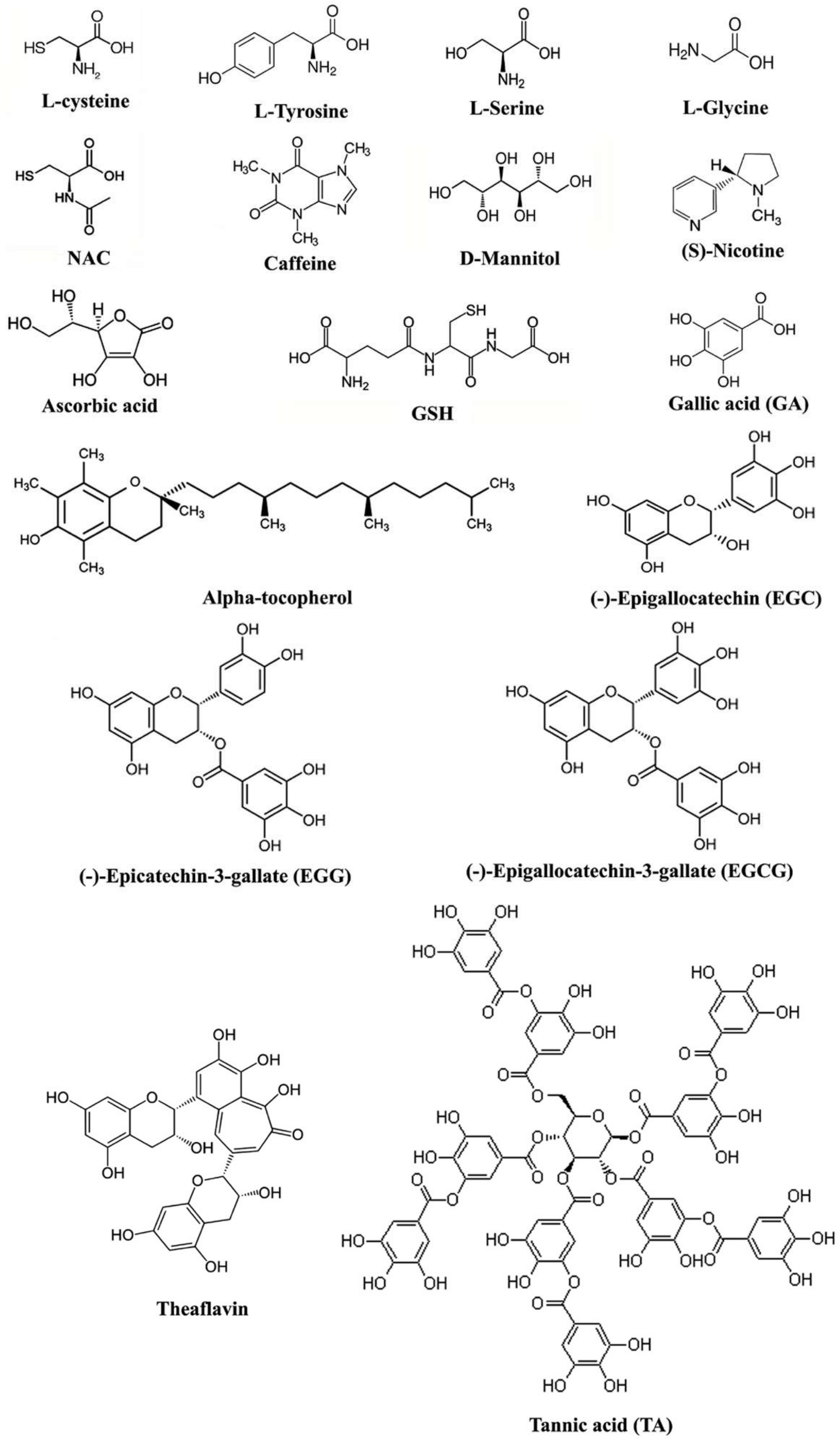

Supplementary Figure S1. Molecular structures of tea polyphenols and other agents.

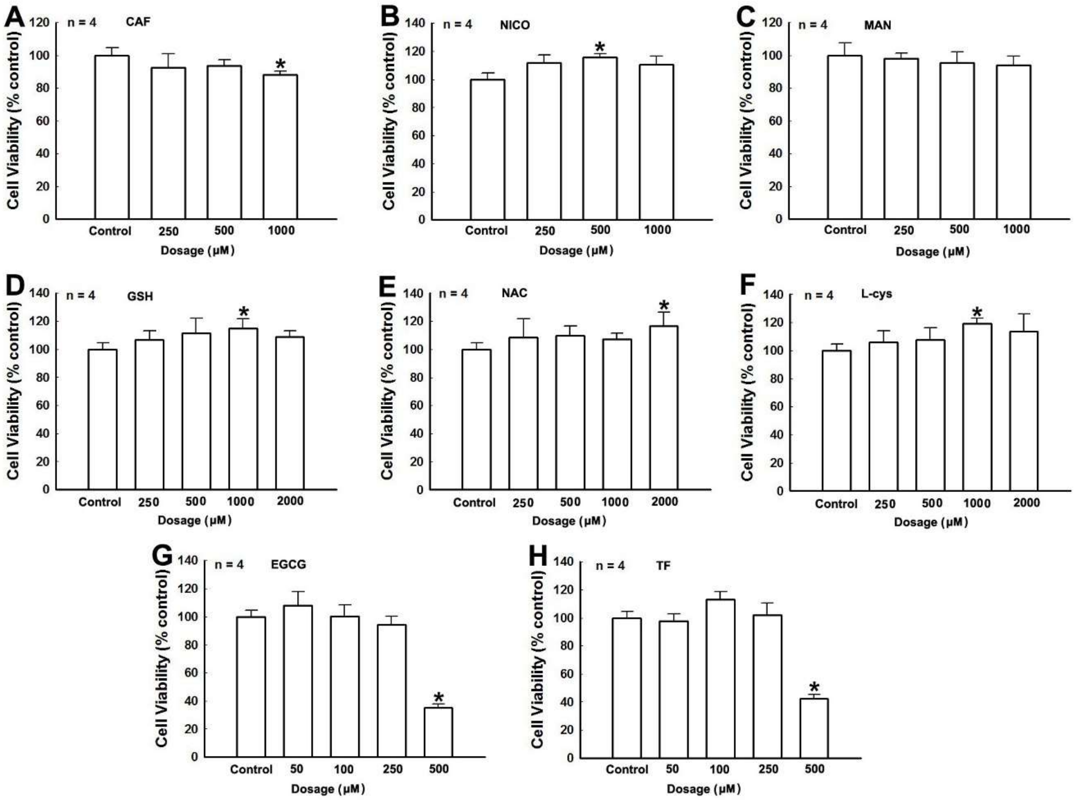

Supplementary Figure S2. Impacts of various agents on cell viability of PC12 cells. Dopaminergic PC12 cells are cultured in the presence or absence of different dosage of agents overnight before monitoring cell viability. Cells without any treatments are set as controls. Cell viability of PC12 treated with agents is expressed as % control. *, $p < 0.001$, compared with cell viability of control cells.

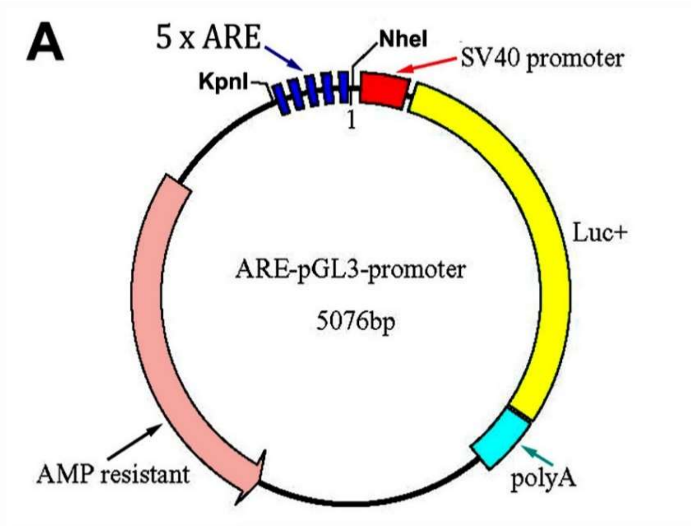

Supplementary Figure S3. Vector constructive map of ARE-pGL3promoter vector.

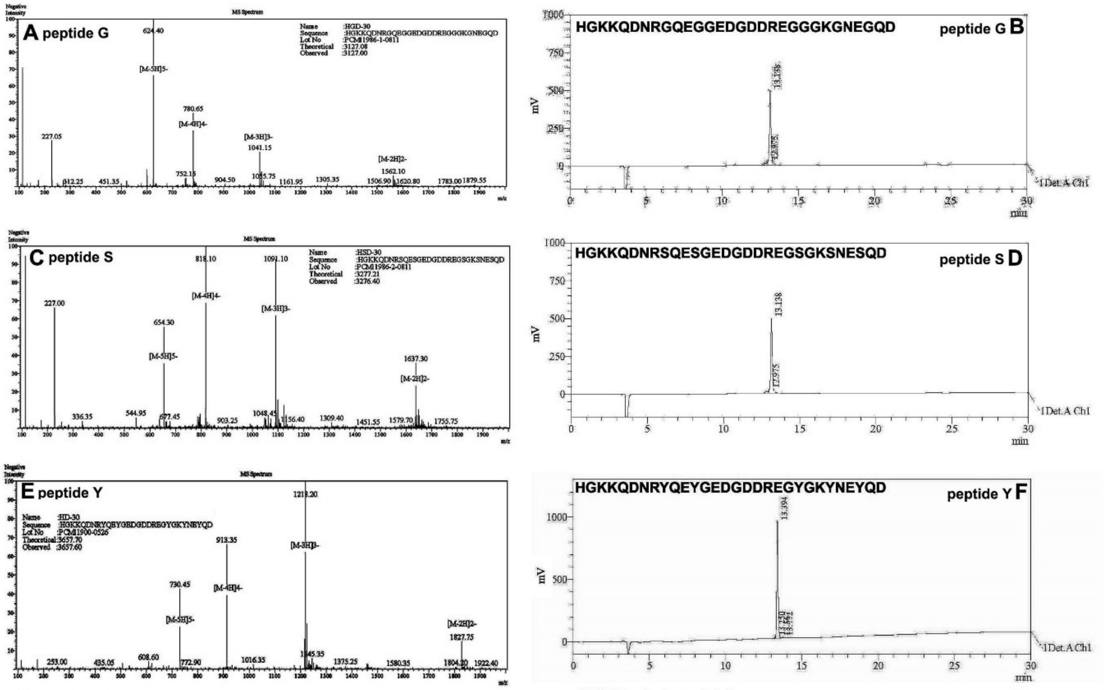

G LC-MS-MS technique details

Dissolution method	:5%NH3.H2O+8%ACN+87%H2O
Date Acquired	: 2016/8/30 9:24:16
Injection Volume	: 3ul
Interface	:ESI
Nebulizing Gas Flow	:1.50L/min
CDL Temp	:250C
CDL Volt	:0v
Block Temp	:200
Prerod Bias	:+4.5kv
Detector	:-0.2kv
T.Flow	:0.2ml/min
B.conc	:50%H2O/50%MeOH

HPLC technique details **H**

Pump A	:0.1% trifluoroacetic in 100% water
Pump B	:0.1% trifluoroacetic in 100% acetonrtrile
Total Flow	:1ml/min
Wavelength	:214nm
Analytical column type	:SHIMADZU Inertsil ODS-SP(4.6*250mm*5um)
Dissolution method	:100%H2O
Inj. Volume	:95ul

Time	Module	Action	Value
0.01	Pumps	B.Conc	0
30.00	Pumps	B.Conc	30
33.00	Pumps	B.Conc	100
38.00	Pumps	B.Conc	100
40.00	Pumps	B.Conc	0
50.00	Controller	Stop	

Supplementary Figure S4. HPLC and LC-MS-MS analysis of 3 peptides synthesized. (**A**,**B**), LC-MS-MS and HPLC chromatographs of peptide G, (**C**,**D**), LC-MS-MS and HPLC chromatographs of peptide S, (**E**,**F**), LC-MS-MS and HPLC chromatographs of peptide Y, (**G**), LC-MS-MS technique details for analysis of peptides, (**H**), HPLC technique details for analysis of peptides.
